# Tirzepatide-Induced Injection Site Reaction

**DOI:** 10.7759/cureus.45181

**Published:** 2023-09-13

**Authors:** Junki Mizumoto

**Affiliations:** 1 Department of Medical Education Studies, International Research Center for Medical Education, Graduate School of Medicine, The University of Tokyo, Tokyo, JPN

**Keywords:** tirzepatide, rash, glucagon-like peptide-1 receptor agonist, diabetes mellitus, adverse drug reaction

## Abstract

A male in his 70s developed a rash on his lower abdomen after changing his subcutaneous injection drug from dulaglutide to tirzepatide. The rash diminished after stopping tirzepatide injection. This case illustrated that tirzepatide can potentially lead to an injection site rash, despite another glucagon-like peptide-1 (GLP-1) receptor agonist (RA) being used without adverse reactions. Injection site reactions are one of the potential adverse events associated with GLP-1 RA use. To the best of our knowledge, this is the first reported case of tirzepatide-induced injection site reaction.

## Introduction

Tirzepatide, a glucose-dependent insulinotropic polypeptide and a glucagon-like peptide-1 (GLP-1) receptor agonist (RA), is a novel drug for diabetes mellitus. Although GLP-1 RA is among the preferred treatment options for individuals with type 2 diabetes mellitus, the increased demand for GLP-1 RA has resulted in global supply constraints, leading to limited availability of dulaglutide and other GLP-1 RAs [1]. In such situations, physicians may be compelled to switch to alternative GLP-1 RAs in some cases. Like other GLP-1 RAs, tirzepatide reportedly has relatively few adverse events. However, the increasing use of tirzepatide may reveal unfounded adverse events. This case illustrated tirzepatide-induced injection site reaction.

## Case presentation

**Figure 1:**
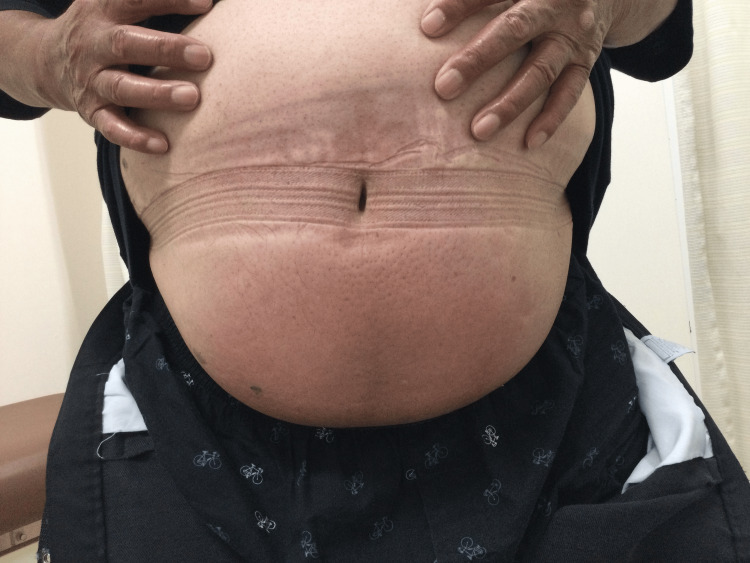
Rash on the lower abdomen 10 days after the initial injection of tirzepatide

A 76-year-old male complained of a rash on his lower abdomen. The patient was a regular patient at our clinic, being managed for obesity, type 2 diabetes mellitus, and alcoholic liver cirrhosis. His regular prescription included subcutaneous dulaglutide 0.75 mg once a week, empagliflozin 25 mg/day, and glimepiride 1 mg/day. Due to a shortage of dulaglutide, the prescription was switched to subcutaneous tirzepatide 2.5 mg once a week. Following the initial injection of tirzepatide into his lower abdomen, the patient developed a rash with soreness at the injection site. The rash progressively enlarged over the subsequent days, causing discomfort characterized by dull pain, burning, and soreness. Consequently, the patient refrained from administering the next injection. Ten days after the first injection, the patient revisited our clinic with a swollen rash that covered the entirety of his lower abdomen (Figure 1). He denied experiencing similar symptoms while on dulaglutide and asserted that his injection technique remained unchanged. The diagnosis of tirzepatide-induced injection site reaction was made. Tirzepatide administration was discontinued, and the skin returned to normal within the following month.

## Discussion

This case illustrated that tirzepatide can potentially lead to an injection site rash, despite other GLP-1 RAs being used without adverse reactions. Injection site reactions are one of the potential adverse events associated with GLP-1 RA use. The risk is minimal for all GLP-1 RAs other than exenatide, but it does exist [2]. The safety profile of tirzepatide aligns with that of the GLP-1 RA class [3,4]. To the best of our knowledge, this is the first reported case of tirzepatide-induced injection site reaction.

The important differential diagnoses include cellulitis and alcohol swab-triggered skin rash. In this case, cellulitis was excluded due to the patient’s appropriate injection technique and the self-limited course of the symptoms. Alcohol swab-induced skin rash typically exhibits a transient and reversible nature, except in instances where the patient is concurrently taking a disulfiram-like medication.

## Conclusions

Tirzepatide may be associated with an injection site reaction, similar to other GLP-1 RAs. The development of a rash due to tirzepatide may occur in patients with no prior history of adverse reactions to other GLP-1 RAs. Physicians should know this adverse reaction to make correct and prompt diagnoses.
